# The Major Histocompatibility Complex in Bovines: A Review

**DOI:** 10.5402/2012/872710

**Published:** 2012-05-28

**Authors:** Jyotsna Dhingra Behl, N. K. Verma, Neha Tyagi, Priyanka Mishra, Rahul Behl, B. K. Joshi

**Affiliations:** Animal Genetics Division, National Bureau of Animal Genetics Resources, P.O. Box 129, GT Bypass Road, Haryana, Karnal 132001, India

## Abstract

Productivity in dairy cattle and buffaloes depends on the genetic factors governing the production of milk and milk constituents as well as genetic factors controlling disease resistance or susceptibility. The immune system is the adaptive defense system that has evolved in vertebrates to protect them from invading pathogens and also carcinomas. It is remarkable in the sense that it is able to generate an enormous variety of cells and biomolecules which interact with each other in numerous ways to form a complex network that helps to recognize, counteract, and eliminate the apparently limitless number of foreign invading pathogens/molecules. The major histocompatibility complex which is found to occur in all mammalian species plays a central role in the development of the immune system. It is an important candidate gene involved in susceptibility/resistance to various diseases. It is associated with intercellular recognition and with self/nonself discrimination. It plays major role in determining whether transplanted tissue will be accepted as self or rejected as foreign.

## 1. Introduction

The major histocompatibility complex (MHC) is a fundamental part of the immune system in nearly all vertebrates [1]. It is one of the most important genetic systems for infectious disease resistance in vertebrates [2, 3]. Therefore, defining the structure, function, and diversity of this system is very important to understand immune response in vertebrate species. There are 3 groups of histocompatibility antigens, class I, class II, and class III. Class I molecules consist of an alpha chain with a molecular mass of about 45 KDa (heavy chain) associated noncovalently with *β*2-microglobulin chain which is around 12 KDa. The class I molecules are expressed in all nucleated cells, and their main function is to present peptides to CD8+ T-lymphocytes, which kill virus-infected and neoplastic cells. Class II molecules are formed by noncovalent association of the *α*- and the *β*-chains encoded by distinct genes within the MHC. Both chains of class II molecules (molecular mass 33 KDa and 28 KDa, resp.) are encoded by genes within MHC. Class II molecules are expressed on “professional” antigen-presenting cells (APCs), such as dendritic cells and macrophages. Class II molecules on APCs present peptides derived from extracellular pathogens to CD4+ T cells, which once stimulated activate macrophages and B cells to generate inflammatory and antibody responses, respectively. The Class III molecules includes products somewhat different from the MHC molecule that too are associated with the immune process, for example, components of complement system, steroid 21-hydroxylase enzymes, and tumour necrosis factors [4].

## 2. Three-Dimensional Structure of the Major Histocompatibility Complex

The three-dimensional structure of class I and class II molecules was determined by X-ray crystallography which revealed sites for peptide binding and interaction with the T-cell receptor for antigen [5–8]. Peptide mapping and amino acid sequencing have revealed that the *α*-chain of the class I MHC molecule is organized into 3 domains *α*1, *α*2, and *α*3. The *α*1 and *α*2 domains are the distal domains that form the antigen (peptide) binding cleft of the class I molecule and the *α*3 domain is the membrane proximal domain. The *α*3 domain and the *β*2-microglobulin bear a significant similarity to each other and to the sequence of the constant region of the immunoglobulins. Each chain in a class II MHC molecule contains two external domains: *α*1and *α*2 domains and *β*1 and *β*2 domains. The *α*1 and *β*1 domains are the distal domains of the class II molecule which form the antigen-binding cleft [9]. The *α*2 and *β*2 domains are proximal to the cell membrane.

## 3. Genomic Organization of the MHC Region

**Figure 1:**
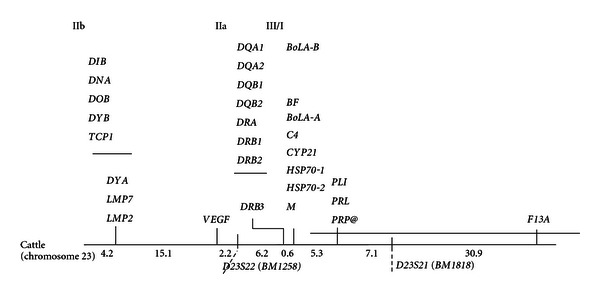
Genetic Linkage map of the major histocompatibility complex region in cattle (World Organisation for Animal Health (OIE)), copied from the Scientific and Technical Review [9].

The bovine MHC genes have been mapped to the bovine autosome 23 (BTA 23) [10, 11] and is referred to as Bovine Lymphocyte antigen (BoLA). This organization distinguishes BoLA from the MHC of humans and rodents wherein the MHC genes are located on chromosome 6 (HLA) and chromosome 17 (H-2 complex), respectively [12]. Physical mapping of bovine MHC was accomplished by Bensaid et al. [13]. The overall size of class I region ranges from 770 Kb to 1650 Kb. There are two tightly linked expressed loci (BoLA-A and BoLA-B) in the BoLA class I region that are within 200 Kb of each other [13]. The class III region is constituted by a heterogeneous set of genes related to immunological and other functions, such as the complement factors BF and C4, steroid 21-hydroxylase (CYP 21), heat shock protein 70 (HSP 70), and tumour necrosis factor *α* and *β* (TNFA and TNFB) [9, 12, 14–19] (Figure 1). The genomic organization of the MHCs of ruminants differs from that of mice and humans as class II region in ruminants is split into two subregions which are separated by at least 15cM (from DYA in the class IIb region to DRB3 in class IIa region) [20, 21]. The class IIa subregion comprises two clusters of genes, DR and DQ. The bovine DQ and DR regions lie in close proximity and are also closely linked to the class III and class I genes, similar in organization to the human orthologues [21, 22]. The DQA and DQB genes have been observed to be duplicated and the duplicated cluster has been observed to be tightly linked in most haplotypes [20]. Data suggest that the organization of class II genes in cattle arose by a chromosomal inversion in an ancestral mammal [23]. Childers et al. [24] identified the inversion breakpoints—comparative analysis with human MHC revealed that the proximal inversion breakpoint occurred approximately 2.5 Kb from the 3′ end of the glutamate-cysteine ligase, catalytic subunit locus, and the distal breakpoint occurred 2 Kb from the 5′ end from a divergent class II DR beta-like sequence. The class IIb region includes the DMA, DMB, LMP2, LMP7, and TAP genes, which are involved in antigen processing and transport [12, 25, 26] and other class II-like genes, such as DNA, DOB, DIB, DYA, and DYB, whose function is unknown [12]. Thus although BoLA genes exist as a system, they are organized in clusters; some loci are tightly linked and others are relatively distant. Significant variations in the recombination rate between different bulls in the interval between DRB3 and DYA have been observed [27, 28]. This suggests the presence of a polymorphic recombination hot spot or insertion or a major chromosomal rearrangement in the region. Further evidence for unusual recombination activity in this interval was obtained from the genetic analysis of secondary oocytes and first polar bodies [29]. On the basis of linkage analysis, bovine class II region lies near the centromere of BTA23, whereas the class I region (BoLA-A locus—the major expressed class I gene) is distal (telomeric) to the class IIa genes [21, 29]. Hybridization of BTA23 with fluorescence labeled DYA and class I probes has demonstrated that these two regions physically map at different positions, with DYA centromeric to class I loci [30]. The BoLA class I genes have not been observed to be separable by recombination [31]. The class III genes lie telomeric to the class IIa region. So far the position of the class III genes with respect to class I genes is not clear—whether they are centromeric or telomeric to the class I genes.

Although studies on this important genetic region have been reviewed previously by many authors, the present paper has been written in an attempt to focus on the class II region of the major histocompatibility complex in bovines namely cattle, buffaloes, yak, and Bison. Bovines are a subfamily in Bovidae family and class mammalia. Bovines include a diverse group of 10 species of medium-sized to large ungulates including domestic cattle, Bison, water buffalo, the yak, and the four-horned and spiral-horned antelopes.

## 4. The Major Histocompatibility Complex Class II Region

The class II region has been characterized using serological techniques [32], cloned heterologous and homologous class II probes [33], RFLP [34, 35], PCR-RFLP [36], and by gene sequencing [37]. Enormous information on the molecular features of the BoLA class II genes can be found in the 8th report of the International Society of Animal Genetics BoLA Nomenclature Committee [25, 26] and on the BoLA website (http://www.ebi.ac.uk/cgi-bin/ipd/mhc/view_nomenclature.cgi?bola.drb3).

## 5. The Major Histocompatibility Complex Class IIa Subregion

### 5.1. DR Genes

The DRA gene encodes the *α*-chain of the DR molecule. BoLA-DRA has been for quite long time considered to be monomorphic since it had only one reported allele. However, some studies lately have shown allelic variation to be present at the bovine DRA locus in cattle. Zhou et al. [38] showed potential sequence variation in the second exon of the bovine DRA, which encodes the antigen-presenting groove. Four unique SSCP patterns were detected among 384 cattle from New Zealand. Sequence analysis revealed that these SSCP patterns represent 4 DRA alleles, and 3 single nucleotide polymorphisms were detected in exon 2.

 Studies on the polymorphism occurring in the DRA locus in buffaloes (Bubu-DRA) were reported by Sena et al., 2003 [39]. Water buffaloes contained two alleles of DRA that differed from each other in two amino acid positions, including one in the peptide binding site. Discovery of variation in DRA was surprising as the first domain of DRA is a highly conserved polypeptide in mammals in general and especially in ruminants.

An and Han [40, 41] submitted two sequences of the variants of the DRA gene in their study on yaks-*Bos grunniens* designated as DRA *01021 (JF298905) and DRA* 01015 (JF298904).

By contrast the genes that encode the *β*-chain of the DR molecule are highly polymorphic. Polymorphism is mainly concentrated in the second exon which encodes the variable portion of the peptide binding site. In cattle there are at least three DRB loci but only one DRB (DRB3) gene is functional. BoLA-DRB1 is a pseudogene with stop codons in the *β*1 and transmembrane region. It shows a very low degree of polymorphism. BoLA-DRB2 is expressed at very low levels if at all. It is monomorphic [42].

#### 5.1.1. Polymorphism at the BoLA-DRB3 Locus in Cattle

The study of the polymorphism of the DRB3 locus attains significance as this region is present in the antigen-presenting site and variability in this region may be related to the variability in the immune responsiveness of different individuals to particular pathogens. The analysis of DRB polymorphism is also useful for inferring the evolutionary history of the MHC in ruminant species [17, 43, 44]. The low level of polymorphism in some wild ruminants such as moose [45, 46] and the considerable differences in allelic frequencies between European and African cattle breeds [47] suggest that selection, genetic drift, and population bottlenecks have played an important role in determining the repertoire of ruminant MHC class I and class II alleles.

Long-range polymerase chain reaction (PCR), cloning, and sequencing were used to define the organization of the DRB3 gene, and estimate the size of the coding region to be approximately 11.4 Kbp that contained the promoter region, DRB3 gene and the 3′ region [48]. BoLA-DRB3.2 which is the second exon of the third DRB bovine gene (DRB3) is responsible for the *β*1 domain of the only widely expressed DRB gene in cattle. A large number of studies carried out to study the genetic variability existing at the DRB3.2 locus in various cattle breeds spread out all over the world have reported the occurrence of a very high degree of polymorphism at this locus and that the allele spectrum and gene frequencies profile of different breeds at this locus vary from each other (Table 1). 

Presently, more than 100 different alleles from exon 2 of the BoLA-DRB3 gene have been identified which parallels the situation for the HLA-DRB1, where more than 290 alleles have been identified [49].

#### 5.1.2. Association of the BoLA-DRB3.2 Alleles with Occurrence of Disease

A number of studies have reported the association of one or more of the BoLA-DRB3.2 alleles with susceptibility/resistance to some infectious diseases in cattle.  Table 2 summarizes the results of different studies carried out to study the association of particular BoLA-DRB3.2 alleles with the resistance/susceptibility to different diseases in different breeds of cattle.

Ballingal et al. [50] studied the association between MHC diversity and the development of bovine neonatal pancytopenia in Holstein Dairy cattle. A comparison of the allelic frequencies between diseased and control groups showed that there was no evidence for any significant differences, suggesting that BoLA-DRB3 does not appear to be a predisposing risk factor in the development of BNP in Holstein dairy cattle. Maillard et al. [51] observed that a unique BoLA class II haplotype made up of one DRB3 exon2 allele and one DQB allele highly correlates with the susceptibility character (*P* < 0.001) to bovine dermatophilosis. This haplotype marker of susceptibility was also found and validated in other bovine populations. A eugenic marker-assisted selection was developed in the field by eliminating only the animals having this haplotype. The disease prevalence was thereby reduced from 0.76 to 0.02 over 5 years. Earlier Lewin et al. [52] had reported that BoLA alleles affect the subclinical progression of bovine leukemia virus infection. This association is strongly correlated with the presence of specific amino acid motifs within the DRB3 antigen-binding domain. Association between BoLA and BLV provides a unique model to study host resistance to retrovirus infection in noninbred species.

#### 5.1.3. Allelic Diversity of DRB3.2 Locus and Vaccination

Studies have also been carried out in a direction to observe if allelic diversity at the class II DRB3 locus plays a role in determining variation to protection by vaccination. Ballingal et al. [53] reported that the bovine Leukocyte Antigen Major histocompatibility complex class II DRB3*2703 and DRB3*1501 alleles are associated with levels of protection against *Theileria parva* challenge following immunization with sporozoite p67 antigen. Glass [54] carried out studies on the genetic variation in MHC and non-MHC genes and the responses to vaccines. A peptide derived from foot- and -mouth disease-virus (FMDV) was used as a probe for BoLA class II function. Both DR and DQ are involved in antigen presentation. In a vaccine trial with several peptides derived from FMDV, BoLA class II DRB3 polymorphisms were correlated with both protection and nonprotection. Although variation in immune responsiveness to FMDV peptide between different individuals is partly explainable by BoLA class II alleles, other genetic factors play an important role. The results suggested that both MHC and non-MHC genes played a role in regulating bovine immune traits of relevance to vaccine design. Baxter et al. [55] reported that the BoLA-DR peptide-binding pockets are fundamental for foot-and-mouth virus vaccine design in cattle. The study investigated associations between bovine major histocompatibility complex DRB3 alleles and their binding pockets with the immune response to a 40 mer peptide derived from foot-and-mouth disease-virus VP1. The amino acids at several positions within the peptide binding cleft of the DR molecule showed significant associations (*P* < 0.001) with the level of antibody response. Further analysis showed that specific residues within the binding pockets are likely to be crucial to vaccine design.

### 5.2. DRB3 Locus in Buffaloes

The chromosomal localization of the major histocompatibility complex in river buffaloes by the technique of fluorescent in situ hybridization (FISH) was first reported by Iannuzzi et al. [56]. The MHC gene was localized to chromosome 2 (BBU2) in river buffalo using the cattle class I cDNA probe. Sumathi et al. [57] used the class II MHC probe, DRB3 clone 11-2 from cattle, to localize the MHC genes bands, to chromosome 2p15-22 by FISH. The study demonstrated that there was a close linkage between class I and class II regions of MHC in buffaloes. Rodrigues Filho et al. [58] mapped the MHC genes in river buffalo. A 5000-rad radiation hybrid mapping panel was generated to elucidate the general organization of the MHC gene on BBU2 and to compare gene order within this region to the MHC in cattle. PCR primers were used from the bovine gene map. Analysis indicated that the class II genes fall into two linkage groups consistent with the organization of the MHC in cattle. Aravindakshan et al. 2000 [59] referred to the MHC gene in buffaloes as BuLA though it is more often referred to as the Bubu-DRB3 gene [60].

#### 5.2.1. Polymorphism at the Bubu DRB3.2 Gene in Buffaloes

Only few studies have been carried out on the polymorphism present at this locus in case of buffaloes [39, 59–65].

Wenink et al. [61] studied the genetic diversity present at the major histocompatibility complex by PCR-RFLP, in the African buffalo, that had experienced bottlenecks, thereby resulting in genetic losses in the population. It was observed that, in spite of the historically known bottlenecks in the population, the MHC-DRB3 gene exhibited surprisingly high level of allelic diversity.

Aravindakshan et al. [59] studied the buffalo MHC DRB3.2 complex polymorphism by the technique of PCR-RFLP in the Murrah and Surti buffaloes. The study resulted in detection of five restriction enzyme patterns namely, “a,” “b,” “d,” “e,” and “i” with the enzyme HaeIII. Digestion of the PCR products with RsaI enzyme in Murrah buffaloes resulted in 10 different restriction digestion patterns while it resulted in 12 different digestion patterns in the case of Surti buffaloes [59]. De et al. [60] studied the polymorphism at the Bubu-DRB3.2 locus by the SSCP and heteroduplex analysis techniques for five Indian riverine buffalo breeds namely, Murrah, Nili-Ravi, Bhadawari, Surti, and Nagpuri. Twenty-two different types of alleles identified in 25 buffaloes indicate a high degree of polymorphism at the DRB locus in this species.

A study on the polymorphism in the MHC-DRB loci of *Bubalus bubalis* identified 6 Bubu-DRB alleles in 19 Murrah animals and 5 Bubu-DRB alleles in 20 Jafarabadi animals [39]. Study of the polymorphism of the DRB3.2 locus by the technique of PCR-SSCP resulted in identification of 14 SSCP patterns in Jaffarabadi buffaloes, 4 SSCP patterns in Mehsani buffalo breed, and 11 SSCP patterns in the Iranian water Azerbaijani buffaloes [62, 64]. Digestion of the BoLA-DRB3.2 locus amplified product by the PstI restriction enzyme resulted in 3 different types of patterns in the Murrah buffalo [63]. The Banni breed of Indian buffaloes was observed to have six alleles, namely, BuLA-DRB3.2*19, *20, *36, *41, *abd, and *gbb in accordance with the nomenclature followed by van Eijk et al. [66] for the BoLA alleles in cattle. The BuLa alleles *abd and *gbb found in the study were new allelic patterns [65].

### 5.3. DRB3 Locus in Bison

American bison (*Bison bison*) and domestic cattle (*Bos taurus *and *Bos indicus*) evolved from a common ancestor 1–1.4 million years ago. They are from different clades of the subtribe Bovina, tribe Bovini, subfamily Bovinae, family Bovidae [67]. The subtribe Bovina has three clades: the first clade includes *Bos taurus *(nonhumped European and African cattle), *Bos indicus* (Indian humped zebu cattle), and *Bison bonasus* (European bison); the second clade includes *Bison bison* (American bison) and *Bos grunniens* (yak); and the third clade includes *Bos frontalis* (gaur) and *Bos javanicus *(banteng) [68, 69]. Morris et al. [70] investigated the polymorphism present at the DRB3 locus in the American bison by the technique of PCR-RFLP and revealed the existence of at least 13 different alleles. Traul et al. [71] characterized the *Bison bison* class IIa haplotypes. The major histocompatibility complex of *Bison bison* is referred to as Bibi corresponding to its counterpart in cattle. The DRB3, DQA, and DQB alleles of Bibi were characterized by PCR-based cloning and sequencing. 12 DRB3 alleles were found to occur in the 14 bison animals studied. Six of the DRB3 alleles were identical to alleles previously identified by Mikko et al. [72]. American bison are highly susceptible to clinical malignant catarrhal fever (MCF). Nevertheless, approximately 20% of bison on ranches or in feedlots become infected with the virus without developing clinical disease. Defining genetic basis for differences in susceptibility between bison could facilitate development of improved control strategies for MCF. DRB3 alleles were classified into resistant (R), susceptible (S), or neutral (N). Animals were classified using six DRB3 genotype categories: N/N, N/R, N/S, R/S, R/R, and S/S. The R/R genotype was associated with resistance to MCF (*P* = 0.0327), while the S/S genotype was associated with clinical MCF (*P* = 0.0069) [5].

Radwan et al. [73] studied the MHC-DRB3 variation in a free-living population of the European bison, *Bison bonasus*. Only four Bibo-DRB3 alleles could be observed in their study which reflected an extreme bottleneck at the beginning of the 20th century.

There is no report so far on the DRB3 locus of the yak-*Bos grunniens mutus*.

#### 5.3.1. DQ Genes

In cattle (Bos taurus), there is evidence of more than 50 alleles of BoLA-DQB (Bovine lymphocyte antigen DQB) that are distributed across at least five DQB loci, making this region one of the most complex in the BoLA gene family. The class II DQ genes of the bovine major histocompatibility complex were investigated by Andersson et al. [74, 75] using human cDNA probes for DQ alpha, DQ beta, DR alpha, and DR beta. The analysis resolved 9 and 12 allelic variants of DQ alpha and DQ beta, respectively. In another study, Andersson and Rask [33] observed that the number of DQ genes varied between haplotypes. Nine DQ_*α*_ alleles designated DQ_*α*_^1^ to DQ_*α*_^9^ and twelve DQ_*β*_ alleles, designated DQ_*β*_^1^ to DQ_*β*_^12^, were distinguished in the study. There was an extreme linkage disequilibrium between the DQ genes since a given DQ_*β*_ allele was always associated with the same DQ_*α*_ allele. The number of DQ genes was found to vary between MHC haplotypes. Four DQ haplotypes, DQ_*α*_^1^_*β*_^1^ to DQ_*α*_^2^_*β*_^4^, possessed a single DQ_*α*_ and a single DQ_*β*_ gene whereas both these genes were duplicated in eight other haplotypes, DQ_*α*_^3^_*β*_^5^ to DQ_*α*_^9^_*β*_^12^. Extensive polymorphism was observed in the first domain exon of bovine DQB genes; the number of bovine DQ genes varied between haplotypes—haplotypes DQ1A, 1B, 2, 3, and 4 contained a single DQA and a single DQB gene. Haplotypes 5–12 contained two DQA and two DQB genes, while haplotype 13 contains two DQA genes and a single DQB gene. The bovine DQB genes were found to be highly polymorphic as ten DQB1 alleles and four DQB2 alleles were identified [76]. The nucleotide sequence of the BoLA-DQA and BoLA-DQB genes was determined and it was observed that the sequences of these genes were very similar to that of the human major histocompatibility complex class II genes [77, 78]. Sequencing and genetic analysis of a bovine DQB cDNA clone by Xu et al. [79, 80] revealed that BoLA-DQ beta-1 has 92% similarity to the coding regions of two previously sequenced BoLA-DQB genomic clones and 69.6% similarity to a Bola-DR beta pseudogene and that the duplication and deletion event that gave rise to DQB1 and DQB2 genes is relatively a recent one in the evolution of cattle MHC. Dikiniene and Aida [81] isolated cDNA clones NB17 and NB25 from cattle lymphoid cell line KU-1 which encoded independent DQB molecules, each of which was a polypeptide of 261 amino acids. The two clones NB25 and NB17 were more closely related to genomic BoLA-DQB clones Y1 and Q1 than they were to each other (90.8% and 93.5% identity at nucleotide and amino acid level, resp.). Polymorphism in the DQB sequences of bovine MHC was investigated in 22 British Friesian cattle. The first domain exon was amplified, cloned, and sequenced. Eight different sequences were identified, six of which had not been reported previously [82]. Expression of multiple DQB genes in *Bos indicus* Kenya Boran cattle was studied by Marello et al. [83]. DQB second exon fragments were amplified by reverse transcription-polymerase chain reaction of total mononuclear cell RNA, cloned, and sequenced. While a single DQB sequence was obtained from some animals, two DQB exon 2 sequences were found in others, implying expression of duplicated DQB genes. A DQB locus which was designated as DQB5 was observed in cattle MHC class II genes. DQB5 which was an additional DQB locus other than the ones already known constitutes an ancient DQB locus which diverged from a common ancestor gene prior to duplication resulting in DQB1 and DQB2 [84]. Maillard et al. [85] sequenced six new BoLA-DQB alleles in the Brahman zebu population of Martinique. Wang et al. [86, 87] identified 36 BoLA-DQB alleles of the DQB-exon2 in 5 breeds of 180 Chinese yellow cattle of which 19 alleles obtained were new.

Nishino et al. [88] isolated and sequenced a new allele of the class II DQA1 gene which was different from the DYA and DRA genes and was most closely related to the sheep BoLA-DQA genes. Morooka et al. [89] isolated cDNA clones encoding the bovine major histocompatibility complex class II DQ alpha chain. One clone MQ9 encoded a primary translated product of 255 amino acids. A new A2 gene in the DQ subregion of the bovine genome was identified from a comparison of amino acid sequences encoded by class II A genes among several species. MQ9 was more closely related to the ovine DQA1 than to the BoLA-DQA2 gene and they represent BoLA-DQA1 genes. Thus, the presence of two BoLA A genes, which may be expressed and functional in the bovine as well as sheep, was confirmed. Fifteen distinct BoLA-DQA1 alleles were observed in a study carried out on 51 animals whose BoLA haplotypes had been characterized at the Fifth International BoLA Workshop by the sequence-based typing method [90]. Class II DQA1 gene was sequenced in Japanese cattle (95 Japanese black, 91 Holstein, 102 Japanese Shorthorn, and 64 Jersey cattle) and the presence of 19 BoLA-DQA1 alleles, of which two were novel alleles, was observed [91]. Holstein, Jersey, Japanese Shorthorn, and Japanese Black breeds had 13, 12, 10, and 15 DQA1 alleles, respectively. Wu-Kabat analysis showed that the DQA1 alleles of the Holstein and Japanese Black were the most and least polymorphic, respectively. Bovidae DQA1 molecule had more polymorphic residues than the human, pig, and dog DQA molecules at two regions, namely, positions 52-53 and 65-66. This indicated that the Bovidae DQA1 locus is more polymorphic than the DQA loci of other species.

Zanotti et al. [92] studied the association of BoLA class II haplotypes with subclinical progression of bovine leukemia virus infection in Holstein-Friesian cattle. Chi-square analysis revealed a significant and absolute association of haplotype DQA*3A-DQB*3A-DRB2*2A-DRB3.2*11 with resistance to persistent lymphocytosis (PL). Animals carrying this haplotype had lower lymphocyte counts (*P* = 0.0057). By contrast, haplotype DQA*12-DQB*12-DRB2*3A-DRB3.2*8 was associated with susceptibility to PL and increased lymphocyte counts (*P* = 0.0537). Gerner et al. [93] carried out study on the identification of major histocompatibility complex restriction and anchor residues of foot and mouth disease-virus derived bovine T-cell epitopes. Their data indicated that epitope located on FMDV protein 1A can be presented by MHC class II DQ molecules encoded by DQA allele 22021 and DQB allele 1301.

#### 5.3.2. DQ Genes in Buffaloes

In buffaloes (*Bubalus bubalis*), Niranjan et al. [94, 95] carried out studies to explore structural and functional variations and possible duplication of the MHC-DQA and DQB gene. Two cDNA sequences amplified from one individual buffalo were designated as Bubu DQA1 (DQA*0101) and-DQA2 (DQA*2001). The percentage of nucleotide and amino acid similarity between Bubu-DQA1 and-DQA2 revealed that these sequences display more similarity to alleles of respective DQA1 and DQA2 genes from other ruminant species than to each other. The phylogenetic analysis also revealed a considerably larger genetic distance between these two genes than between homologous genes from other species. The larger genetic distance between DQA*0101 and DQA*2001 and the presence of different bovine DQA putative locus specific amino acid motifs suggests these sequences are nonallelic. So as in the case of other ruminants there is a duplication of the DQA gene in buffaloes as well. When genetic diversity in the DQA and the DQB genes was studied a total of 7 and 10 alleles were identified for DQA and DQB loci, respectively. Nucleotide and amino acid variations among DQ alleles at peptide-binding sites were high. Variations were higher in DQB than DQA alleles. Sena et al., 2011 [96] studied 3 breeds of water buffalo, namely Murrah, Jafarabadi, and Mediterranean and one breed of swamp buffalo (Carabao) in Brazil and observed 12 Bubu-DQB alleles in these buffalo breeds.

#### 5.3.3. DQ Genes in Bison

In *Bison bison* [71] 11 Bibi-DQA and 10 Bibi-DQB alleles were reported. For each bison class II allele, it was possible to identify closely related cattle sequences. Also, Bison haplotypes with both nonduplicated and duplicated DQ genes were identified. These haplotypes appeared to have originated from the same ancestral haplotypes as orthologous cattle haplotypes.

### 5.4. The Major Histocompatibility Complex Class IIb Subregion

In humans, the DMA and DMB genes encode a molecule that plays a role in the complexing of peptides with class II molecules [97], whereas DNA and DOB encode a protein that might regulate the function of the DM molecule [98]. The DMA/DMB and DNA/DOB orthologues have been identified in cattle [9, 99]. Van der Poel [77] studied the nucleotide sequence of the bovine DRA, DOA, and DYA genes. No clear homologue of the BoLA-DYA gene was observed to be present among the human class II genes; it appears to be a distinct bovine class II gene. Another bovine class II *β*-chain gene, BoLA-DIB, also has a nucleotide sequence distinct from DRB/DQB genes. This DIB gene is tightly linked to DOB, suggesting that DIB is in this second group of bovine class II genes [100, 101]. Southern blot analysis showed that a gene similar to BoLA-DIB is present in several representative members of the Bovidae family as well as the Cervidae and Giraffidae families. Davies et al. [32] studied the polymorphism of the DRB, DQA, DQB, DYA, DOB, and DIB genes by the technique of PCR-RFLP. Five class IIb (DYA-DOB-DIB) haplotypes were defined. Shalhevet et al. [102] reported that the LMP2 proteasome subunit gene was present in the BoLA class IIb region. Gelhaus and Forster [103] reported that the loci DOA and DOB could be genetically mapped to the Bola class IIb region by linkage analysis. They studied the polymorphism present across the second exons of the MHC DOA and DOB loci and could detect two and four allelic variants respectively. In the predicted amino acid sequence, the DOA polymorphism corresponded to the variation at the respective residue position, whereas the nucleotide substitutions in the DOB gene were noninformative. Ballingal et al. [53] reported that the BoLA DYA and DIB genes in cattle formed a closely linked pair characteristic of other class II MHC molecules. Therefore, it was suggested that as per accepted nomenclature conventionally BoLA-DIB should be renamed BoLA-DYB. The full-length DYA and DYB transcripts revealed open reading frames with potential to translate 253 and 259 amino acid proteins, respectively. Comparative sequence analysis between the DY polypeptides and classical cattle, human and mouse MHC alpha and beta polypeptide chains revealed 16 unique amino acid residues at positions predicted to form and line the putative peptide-binding region. The function of the DYA and DYB genes is however unknown [104]. As the functional genes show higher levels of nucleotide conservation than the pseudogenes, the DYA genes from sheep and cattle were compared—the two species diverged from each other approximately 20 million years ago. Levels of conservation in the immediate promoter, coding region, and intronic regions were 97%, 94%, and 91% respectively which were comparable to that of the functional MHC genes. The degree of conservation between these class II MHC genes is consistent with evolution under purifying selection suggesting that these genes retain a unique function in ruminants.

### 5.5. Binding of Peptides to Class II Major Histocompatibility Complex

Srinivasan et al. [105] showed that very few molecules of the MHC class II receptors per antigen-presenting cell bind processed antigen and this number is sufficient for T-cell activation. In another study Chicz et al. [106] reported the characterization of the acid-eluted peptides bound to HLA-DR1 by high-performance liquid chromatography, mass spectrometry, and microsequencing analyses. The relative molecular masses of peptides varied between 1602 and 2996 (13–25 residues). In most abundant individual the molecular weight values ranged between 1700 and 1800 that corresponded to an average peptide length of 15 residues. Peptides bound to class II molecules may have some related features. Mouritsen et al. [107] carried out studies on the characterization of the MHC class II-bound self-peptides by separating them by isoelectric focusing. They observed that the peptides eluted from one allelic form of MHC class II molecule did bind to MHC class II molecules of the same allelic form, but not to MHC class II molecules of a different form.

Study on the characterization of the peptide bound to the MHC class II molecule attains importance as this shall help in understanding better the mode of its action and also in the formulation of the vaccines against a particular disease.

## 6. Conclusion

 Study of the major histocompatibility complex assumes importance because of the critical role it plays in the immune system of the animal. The extensive structural polymorphism of class II molecules is responsible for the differences among individuals in immune response to infectious agents. This high degree of polymorphism observed at the DRB3.2 locus may help in identification of superior haplotypes for disease resistance. Also the study of the MHC can aid in the development and the design vaccines based on synthetic peptides comprising of one or more T-cell epitopes of the pathogen.

## Figures and Tables

**Table 1:** Different BoLA-DRB3.2 alleles and the alleles with gene frequency higher than 0.05 found at the DRB3.2 locus in different cattle breeds.

Breed	*n* ^a^	*n* ^a^_a_	%^a^	Alleles with gene frequency higher than 0.05	*N*
Holstein^b,c,d^	27	7	88.7	DRB3.2*03, *08, *11, *16, *22, *23 and *24	835
	22	6	71	DRB3.2*08, *11, *23, *22 and *16	127
	29	6	70.3	DRB3.2*22, *24, *08, *16, *23 and *11	1100
Jersey^b,e^	13	7	82.4	DRB3.2*07, *10, *17, *21, *20, *28 and *32	66
	24	6	74	DRB3.2*08, *10, *15, *21, *36 and *ibe	172
Saavedreno^f^	22	7	70	DRB3.2*16, *36, *08, *11, *27, *37 and *07	125
Argentine^g^	21	6	72.8	DRB3.2*05, *15, *18, *20, *24 and *27	194
Japanese Shorthorn^h^	21	6	70	DRB3.2*08, *09, *21, *27, *07 and *24	176
Russian Ayrshire^i^	18	5	77	DRB3.2*07, *28, *08, *10 and *24	127
Iranian Holstein^j^	26	4	67	DRB3.2*08, *24, *11, *16	250
Iranian Golpayegani^k^	19	9	74	DRB3.2*52, *45, *28, *19, *16, *11 and *10	50
Norwegian Red^l^	27	7	78.1	DRB3.2*03, *07, *08, *11, *24, *26, *27	523
Kankrej^m^	24	6	71	DRB3.2*15, *06, *20, *37, *46, *34	50
Sahiwal^n^	20	6	67	DRB3.2*02, *15, *08, *09, *37	
Rathi^n^	13	5	68	DRB3.2*10, *15, *08, *09 and *37	51
Hariana^n^	16	5	59	DRB3.2*02, *06, *08, *20 and *36	35
Tharparkar^o^	15	5	62	DRB3.2*01, *37, *taa, *i^1^cc and *10	33
Iranian Holstein^p^	28	6	69.65	DRB3.2*08, *11, *16, *22, *23 and *24	262
Chinese Yellow^q^	23	7	53.9	DRB3.2*2002, *2003, *3101, *3103, *4302, *5702, *6001	80
Iranian Sistani^r^	32	6	60	DRB3.2*08, *10, *11, *20, *34 and *X	65
Japanese Holstein^s^	16	4	56.8	DRB*0101, *1501, *1201 and *1101	194
Japanese Holstein^t^	17	6		DRB*0101, *1001, *1101, *1201, *1501 and *2703	143
Japanese Black^t^	22	7		DRB3*0201, *0503, *1001, *1101, *1201, *1501 and *1601	507

*n* ^a^-Total number of alleles in the breed studied, *n* ^a^_a_number of alleles with frequency higher than 0.05*N*-Total number of animals studied, ^b^Sharif et al. [108], ^c^Dietz et al. [109], ^d^Dietz et al. [110], ^e^Gilliespie et al. [111], ^f^Ripoli et al. [112], ^g^Giovambattista et al. [113], ^h^Takeshima et al. [114], ^i^Udina et al. [115], ^j^Nassiry et al. [116], ^k^Mosafer and Nassiry [117], ^l^Kulberg et al. [118], ^m^Behl et al. [119], ^n^Behl et al. [120], ^o^Behl et al. [121], ^p^Pashmi et al. [122], ^q^Wang et al. [123], ^r^Mohammadi et al. [124], ^s^Yoshida et al. [125], ^t^Miyasaka et al. [126].

**Table 2:** Association of the BoLA-DRB3.2 alleles with different diseases in different breeds of cattle.

Disease	Breed	BoLA allele	Type of association (resistance/susceptibility)
Dermatophilosis	Brahman^a'^	BoLA-A8, BoLA-DRB3 “EIAY”	Higher resistance
Severe mastitis	Canadian Holstein^b'^	BoLA-DRB3.2*23	Higher susceptibility
Retained placenta	Canadian Holstein^b'^	BoLA-DRB3.2*03	Lower susceptibility
Subclinical mastitis	Iranian Holstein^c'^	BoLA-DRB3.2*08	Higher susceptibility
Tick infestation by Amblomma americanum	*Bos taurus *x *Bos indicus * ^d'^	DRB3*4401	Resistance
Clinical mastitis	Canadian Holstein^e'^	DRB3.2*03, DRB3.2*11	Lower susceptibility
Clinical mastitis	Canadian Holstein^e'^	DRB3.2*08	Higher susceptibility
Clinical mastitis	Norwegian Red^f'^	DRB3.2*13, *18, *22 and *27	Lower susceptibility
Mastitis	Japanese Holstein^g',h'^	DRB3*0101, *1501	Susceptibility
Mastitis	Japanese Holstein^g',h'^	DRB3*1101, *1401, *1201	Resistance
Lymphosarcoma and persistent lymphocytosis caused by Bovine Leukemia virus	Holstein^i'^	BoLA DRB3.2*11 subtype ISAG*0902	Resistance
Leukemia	Russian Black Pied^j'^	BoLA-DRB3.2*11, *23 and *28	Resistance

^a'^Maillard et al. [127], ^b'^Sharif et al. [108], ^c'^Pashmi et al. [122], ^d'^Untalan et al. [128], ^e'^Rupp et al. [129], ^f'^Kulberg et al. [118], ^g',h'^Yoshida et al. [125, 130], ^i'^Juliarena et al. [131], ^j'^Sulimova et al. [132].
